# Channel Estimation for Massive MIMO Systems via Polarized Self-Attention-Aided Channel Estimation Neural Network

**DOI:** 10.3390/e27030220

**Published:** 2025-02-21

**Authors:** Shuo Yang, Yong Li, Lizhe Liu, Jing Xia, Bin Wang, Xingjian Li

**Affiliations:** 1 54th Research Institute of China Electronics Technology Group Corporation, Shijiazhuang 050081, China; yang_shuo2025@126.com (S.Y.); xxbzdh@163.com (L.L.); xiajing_2024@163.com (J.X.); ctiwangbin1888@hotmail.com (B.W.); lixingjianjan@163.com (X.L.); 2 National Key Laboratory of Advanced Communication Networks, Shijiazhuang 050081, China

**Keywords:** massive MIMO, channel estimation, polarized self-attention-aided channel estimation network

## Abstract

Research on deep learning (DL)-based channel estimation for massive multiple-input multiple-output (MIMO) communication systems has attracted considerable interest in recent years. In this paper, we propose a DL-assisted channel estimation algorithm that transforms the original channel estimation problem into an image denoising problem, contrasting it with traditional experience-based channel estimation methods. We establish a new polarized self-attention-aided channel estimation neural network (PACE-Net) to achieve efficient channel estimation. This approach addresses the limitations of the conventional methods, particularly their low accuracy and high computational complexity. In addition, we construct a channel dataset to facilitate the training and testing of PACE-Net. The simulation results show that the proposed DL-assisted channel estimation algorithm has better normalization mean square error (NMSE) performance compared with the traditional algorithms and other DL-assisted algorithms. Furthermore, the computational complexity of the proposed DL-assisted algorithm is significantly lower than that of the traditional minimum mean square error (MMSE) channel estimation algorithm.

## 1. Introduction

Massive multiple-input multiple-output (MIMO) technology represents a pivotal advancement in the fifth generation of mobile communications and is anticipated to serve as a significant enabling technology for the sixth generation. In comparison to single-input single-output systems, massive MIMO systems exhibit superior spectral efficiency, thereby improving the user experience within the constraints of the available spectrum resources [1]. To fully leverage the exceptional performance of massive MIMO systems, precise channel state information (CSI) is essential. Thus, the development of accurate physical-layer channel estimation algorithms is of paramount importance in the context of massive MIMO systems.

Traditional massive MIMO channel estimation techniques can be classified into three categories: pilot-based channel estimation [2], blind channel estimation, and semi-blind channel estimation. Among these, the pilot-based channel estimation technique reconstructs the channel response by utilizing a priori channel information, resulting in enhanced accuracy and reliability. This approach has been extensively studied and employed by researchers. The commonly used pilot-based channel estimation algorithms include least square (LS) estimation [3] and minimum mean square error (MMSE) estimation [4]. The LS channel estimation algorithm is characterized by its lower computational complexity. However, it does not account for the influence of noise. It relies on the error between the actual observations and the estimated observations, which makes it particularly susceptible to noise enhancement in practical applications. This susceptibility is especially pronounced when the channel experiences large-scale fading, leading to significant degradation in the accuracy of channel estimation. To address the limitations of the LS channel estimation algorithm, researchers have proposed the MMSE channel estimation algorithm. This algorithm directly utilizes the error between the true value of the estimator and the estimated value as the optimization objective. This approach effectively reduces the influence of noise, resulting in more accurate channel estimation in comparison to the LS method. Thus, the complexity of the algorithm increases dramatically with the increase in the number of antennas in massive MIMO communication systems, thereby presenting challenges for its effective implementation in practical engineering contexts. To reduce the computational complexity associated with the MMSE channel estimation algorithm, some researchers have introduced the linear minimum mean square error (LMMSE) channel estimation algorithm. This approach incorporates a linear smoothing process based on MMSE channel estimation and eliminates one matrix inverse operation and utilizes expected values instead of true values. This modification sacrifices some algorithm performance in exchange for lower computational complexity. In recent years, channel estimation algorithms based on compressive sensing (CS) have been proposed [5] and integrated with sparse structures to obtain channel state information by using the joint orthogonal matching pursuit (OMP) algorithm [6,7]. However, a channel estimation algorithm based on CS is heavily reliant on channel sparsity, necessitating that the channel exhibit pronounced sparsity characteristics. It is not appropriate for non-sparse channels.

The rapid advancement in computing resources has facilitated significant developments in artificial intelligence, particularly in deep learning (DL) technologies. These advancements have led to revolutionary breakthroughs characterized by exceptional performance in various domains, including computer vision, speech signal processing, and natural language processing. In light of this, some researchers have begun to explore the integration of artificial intelligence theories and methodologies within the domain of wireless communications to establish intelligent wireless communication systems that synergize traditional wireless communication technologies, thereby achieving novel advancements in the field [8]. Furthermore, massive MIMO technology leverages an ultra-high antenna configuration to optimize spatial resources, significantly enhancing the capacity of communication systems. This advancement also generates substantial volumes of wireless communication data, providing essential support for the application of artificial intelligence technologies in wireless communication.

The current research in the domain of intelligent wireless transmission technology predominantly focuses on the application of DL-based algorithms as a substitute for conventional wireless transmission algorithms. This focus is evident in the implementation of deep learning techniques aimed at achieving objectives such as intelligent channel estimation [7,8,9,10,11,12,13,14], signal detection [15,16,17,18], CSI feedback [19,20,21,22], and channel coding [23,24]. At present, the majority of the research in intelligent channel estimation employs DL-based methods that conceptualize the channel matrix as a two-dimensional image, thereby transforming the channel estimation challenge into an image denoising problem within the realm of computer vision. For instance, the study presented in [7] explores DL-based sparse channel estimation techniques in a frequency division duplex (FDD) mode for massive MIMO systems. The author proposes a ConCSNet neural network (NN) architecture, which effectively reconstructs the original sparse channel. However, this algorithm necessitates a certain level of channel sparsity. The research detailed in [13] addresses the channel estimation problem in orthogonal frequency division multiplexing (OFDM) systems, introducing a dual-data and model-driven channel estimation algorithm. This algorithm demonstrates a performance advantage over the LMMSE algorithm at low signal-to-noise ratios (SNRs) below 0 dB. However, its performance is largely comparable to that of the LMMSE algorithm at SNRs exceeding 0 dB. Additionally, the work in [25] investigates DL-based channel estimation techniques for massive MIMO-OFDM communication scenarios, achieving estimation performance that is fundamentally comparable to that of the MMSE algorithm. Furthermore, the study in [26] introduces CBDNet, a two-stage NN model comprising a noise intensity estimation subnetwork and a non-blind denoising subnetwork. This model employs an asymmetric joint loss function for training, resulting in accelerated convergence and superior estimation performance relative to traditional channel estimation algorithms. Nonetheless, the model exhibits high computational complexity. Further, its channel estimation performance is heavily reliant on the accurate estimation of noise intensity by the noise intensity estimation subnetwork, which also contributes to relatively poor model stability. Moreover, the research in [27] utilizes self-encoders to jointly train the pilot signal and channel estimation network, thereby enhancing channel estimation performance in downlink massive data MIMO systems. The work presented in [28] proposes an attention mechanism that leverages channel distribution properties, demonstrating the effectiveness of this mechanism in improving the performance of channel estimation algorithms. Lastly, the study in [29] integrates imperfect channel estimation for massive MIMO-OFDM into a deep neural network (DNN) framework, effectively replacing interpolation methods and achieving enhanced channel estimation accuracy.

In conclusion, channel estimation algorithms utilized in massive MIMO communication systems face significant challenges in terms of diminished algorithmic accuracy, heightened computational complexity, and stringent requirements regarding channel sparsity. To address these challenges, we explore a DL-assisted intelligent channel estimation technique that integrates traditional channel estimation algorithms with NN models. The primary contributions of this paper are summarized as follows:

(1) We consider the issues of low estimation accuracy and high computational complexity associated with traditional channel estimation algorithms. We investigate a DL-assisted massive MIMO channel estimation algorithm and introduce a novel DNN model. This algorithm integrates traditional channel estimation algorithms and NN models, enhancing estimation performance while simultaneously reducing computational complexity. In addition, the algorithm does not necessitate the sparsity of the channel. This is achieved through the implementation of data-driven approaches, as well as offline training and online testing methodologies.

(2) Based on a deep learning algorithm, the polarized self-attention-aided channel estimation neural network (PACE-Net) is proposed. Combining the ideas of a residual NN and polarized self-attention mechanism, PACE-Net offers several advantages: (1) enhanced feature extraction capabilities, enabling more profound analysis of both noise and effective signal features, which results in improved channel estimation accuracy. (2) The incorporation of a residual structure mitigates the risk of feature loss, thereby enhancing the scalability of the model. (3) The focus on noise feature extraction, as opposed to solely effective signal feature extraction, reduces the number of layers within the NN model, contributing to its lightweight design. (4) PACE-Net can be flexibly integrated with traditional channel estimation algorithms, facilitating further performance enhancements.

(3) A method for producing a massive MIMO channel dataset for training and testing of NNs is proposed. To mitigate the limitations of NNs in processing complex data, the complex channel matrix is transformed into a real channel matrix. This transformation avoids the drawbacks of NNs when handling complex data directly while preserving essential channel information. Furthermore, to mitigate potential information loss that may occur when converting a two-dimensional matrix into a grayscale image, the proposed method utilizes the two-dimensional channel matrix directly as the input to the NN.

(4) Substantial simulations are provided in this paper to analyze and elucidate the performance of PACE-Net. The results indicate that PACE-Net achieves a superior normalization mean square error (NMSE) performance when compared to traditional algorithms and other NNs. Furthermore, the computational complexity of the DL-assisted channel estimation algorithm is significantly lower than that of the traditional MMSE channel estimation algorithm.

The notation employed in this paper is delineated as follows: scalars, vectors, and matrices are represented using italics, lowercase bold, and uppercase bold, respectively. The symbols $X^{T}$, $X^{H}$, and $X^{-1}$ denote the transpose matrix, the Hermitian conjugate matrix, and the inverse matrix of a matrix X, respectively. The notation $C^{x\times y}$ signifies the complex space of dimensions x×y. The expression x∼CN(μ,Σ) indicates that the random vector x follows a circularly symmetric complex Gaussian distribution with mean μ and covariance matrix Σ. The operator vec(X) represents the vectorization of the matrix X, while $\overset{\Delta }{=}$ indicates a definition, and ⊗ denotes a definition. The symbol ⊗ indicates the Kronecker product of matrices. The notation O(•) refers to the time complexity of the algorithm. The expression ${∥•∥}_{F}$ denotes the Frobenius norm, while ${∥•∥}_{2}$ represents the $l_{2}$ norm, and *E* denotes the mathematical expectation.

## 2. System Model

**Figure 1:**
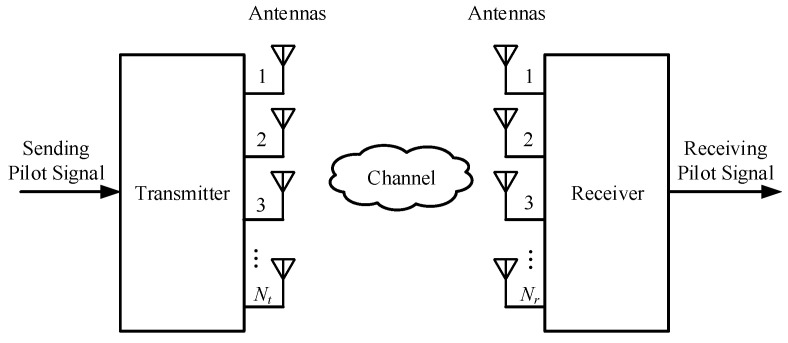
Block diagram of massive MIMO communication system structure.

Consider a channel estimation problem in a massive MIMO system where both the transmitter and receiver are equipped with antenna arrays comprising multiple antennas, as illustrated in Figure 1. The transmitter transmits an orthogonal pilot sequence ${\left\{x_{t}\right\}}_{t=1}^{T}$ for channel estimation. This sequence is subsequently transmitted to the receiver after traversing the channel represented by $H\in C^{N_{r}\times N_{t}}$. Consequently, the received signal at the receiver during time slot *t* can be articulated as follows:

(1) $$y_{t}=Hx_{t}+w_{t},\;t=1,2,\ldots ,T,$$

where $N_{t}$ and $N_{r}$ represent the number of antennas at the transmitter and receiver, respectively. The term $w_{t}∼\mathrm{CN}\left(0,\sigma_{n}^{2}I\right)$ denotes additive complex Gaussian white noise characterized by a zero mean and variance $\sigma_{n}^{2}$. The variable *T* indicates the number of pilots. The aggregation of the received signals from the pilots over the entire time slots is represented in Equation (2). Furthermore, the vectorization of both the left and right sides is articulated in Equation (3).

(2) $$\begin{array}{cc} Y & =\left[y_{t},\ldots ,y_{T}\right]\\ & =H\left[x_{1},\ldots ,x_{T}\right]+\left[\begin{array}{c} w_{1},\ldots ,w_{T} \end{array}\right]\\ & =HX+W, \end{array}$$

(3) $$\begin{array}{cc} y & =\mathrm{vec}(Y)\\ & =\left[X^{T}⊗I\right]\mathrm{vec}\left(H\right)+\mathrm{vec}\left(W\right)\\ & =\Phi h+w. \end{array}$$

To mitigate the computational complexity associated with channel estimation, we employ mutually orthogonal pilot sequences, represented mathematically as $XX^{H}=pI$, where *p* signifies the power of the pilot signal transmitted by the sender. In this context, orthogonal pilot sequences can be achieved by selecting the columns of an orthogonal matrix, such as a discrete Fourier matrix.

Channel modeling serves as a foundational element for the examination of massive MIMO channel estimation techniques. In the realm of wireless communications, various approaches to massive MIMO channel modeling are employed, including the Rayleigh channel model, the Kronecker channel model, the Rice channel model, the tapped delay line channel model, the clustered delay line channel model, the spatial channel model, the link-level channel model, the intelligent multiple transmitter multiple receiver antenna channel model, and the Saleh–Valenzuela channel model, among others. In massive MIMO wireless communication systems, it is essential to formulate the correlation between antennas. Accordingly, we utilize the Kronecker channel model for channel representation [30], and the channel can be expressed as

(4) $$H=R_{\mathrm{Rx}}^{\frac{1}{2}}H_{w}R_{\mathrm{Tx}}^{\frac{1}{2}},$$

where $H_{w}$ represents a Rayleigh fading channel characterized by mutually independent channel coefficients. To model the correlation matrices $R_{\mathrm{Rx}}$ and $R_{\mathrm{Tx}}$ between the antennas, the classical exponential correlation model is employed. Specifically, the entries located in the *i*-th row and *j*-th column of matrices $R_{\mathrm{Rx}}$ and $R_{\mathrm{Tx}}$ are formulated as

(5) $$R_{\mathrm{Rx}}\left(i,j\right)=R_{\mathrm{Tx}}\left(i,j\right)={\tilde{a}}^{d_{i,j}}\overset{\Delta }{=}{\tilde{a}}^{d_{0}c_{i,j}}\overset{\Delta }{=}a^{c_{i,j}},$$

where $d_{i,j}\geq 0$ denotes the distance from the *i*-th antenna to the *j*-th antenna, $c_{i,j}≜d_{i,j}/d_{0}$ denotes the spacing between neighboring antennas, $c_{i,j}≜d_{i,j}/d_{0}$ is a constant, and $\tilde{a}\in \left\lceil 0,1\right\rceil$ and $a\in \left[\begin{array}{c} 0,1 \end{array}\right]$ are both parameters. Based on the established model, it can be inferred that the vectorized representation h=vec(H) of the channel matrix H follows a complex Gaussian distribution:

(6) $$h∼CN\left(0,R_{h}\right),$$

and the covariance matrix of the channel is

(7) $$R_{h}=\delta^{2}\left(R_{\mathrm{Tx}}⊗R_{\mathrm{Rx}}\right),$$

here, δ is the large-scale fading of the channel. In the context of channel estimation, given the pilot matrix X and the pilot received signal y, the objective is to estimate the channel represented by y. This involves deriving the vector y from the matrix Φ and the vector h.

## 3. Introduction to Channel Estimation Algorithms for Massive MIMO Communication Systems Based on LS and MMSE

Initially, a brief review of classical LS- and MMSE-based channel estimation algorithms is presented. The LS-based channel estimation algorithms employ the least squares criterion as the optimization objective to estimate the channel as follows:

(8) $${\hat{h}}_{\mathrm{LS}}={\left(\Phi^{H}\Phi \right)}^{-1}\Phi^{H}y.$$

The expression for the MMSE channel estimation algorithm is provided by

(9) $${\hat{h}}_{\mathrm{MMSE}}=R_{h}\Phi^{H}{\left(\Phi R_{h}\Phi^{H}+\sigma_{n}^{2}I\right)}^{-1}y.$$

It is important to note that the computational complexity of the LS channel estimation algorithm can be reduced to linear complexity when the matrix Φ is orthogonal. In this scenario, the computational complexity of the MMSE channel estimation algorithm is significantly greater than that of the LS channel estimation algorithm, primarily due to the requirement for matrix inversion.

The LS-based channel estimation algorithm exhibits suboptimal performance under low-SNR conditions, primarily due to its failure to adequately account for the effects of noise. As a result, there is considerable potential for performance improvement. Conversely, the MMSE-based channel estimation algorithm offers greater accuracy than the LS algorithm. However, its computational complexity escalates significantly with an increasing number of antennas, primarily due to the matrix inversion operation. Furthermore, while multiple antenna arrays provide additional gain, they also impose greater demands on the performance and computational complexity of channel estimation algorithms owing to the substantial increase in the number of channel coefficients that require estimation. In contrast to these traditional approaches, we propose a DL-assisted channel estimation method based on PACE-Net. This method facilitates a nonlinear channel estimation process that is learned from channel data in a data-driven manner, thereby enhancing algorithmic performance. Furthermore, the DL-assisted framework proposed here utilizes an offline training and online testing paradigm, resulting in a significant reduction in computational complexity when compared to conventional channel estimation algorithms.

## 4. Channel Estimation Algorithm for Massive MIMO Communication Systems Based on Polarized Self-Attention-Aided Channel Estimation Neural Network

### 4.1. Algorithm Design

**Figure 2:**
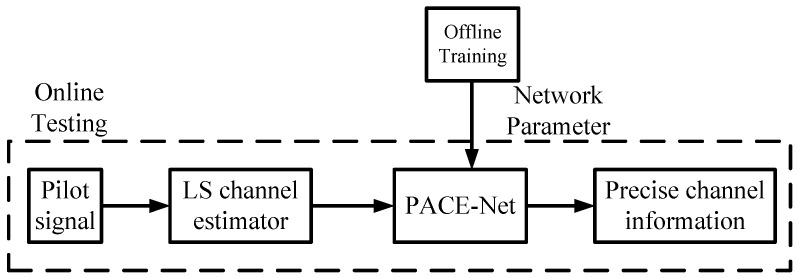
Flowchart of a channel estimation algorithm utilizing a polarized self-attention-assisted neural network.

The process of the DL-assisted intelligent channel estimation algorithm is illustrated in Figure 2. The algorithm is categorized into two distinct phases: offline training and online testing. In the offline training phase, the proposed PACE-Net is applied to a training dataset, where it undergoes training in an offline manner. Then, the trained model parameters need to be preserved. During the online testing phase, the receiver estimates the initial value based on the received pilot information utilizing the traditional channel estimation algorithm. This initial estimate is then input into PACE-Net, which is initialized with the parameters saved during the offline training phase. PACE-Net processes the input data to produce an accurate channel estimate.

DL networks characterized by deeper architectures and an increased number of parameters are capable of comprehensively learning the intricate mapping relationships between network inputs and target outputs. The DL networks proposed in this paper effectively learn the distribution of noisy data, extract relevant features from this noisy information, and subsequently eliminate the extracted noise from the channel information through a data training process. This approach yields more accurate channel estimation results. Furthermore, the DL network autonomously learns the distribution of noisy data during the training phase through a data-driven methodology, which does not impose specific requirements on the channel characteristics. Consequently, it is not necessary for the channel to exhibit a certain degree of sparsity.

### 4.2. PACE-Net Model Architecture

**Figure 3:**
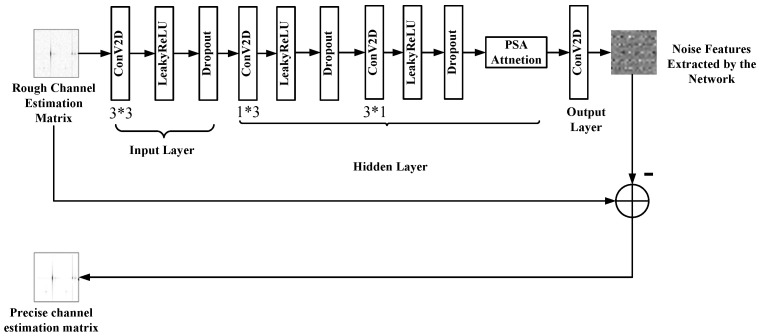
PACE-Net model structure diagram.

The architecture of the PACE-Net model is illustrated in Figure 3. It is inspired by the design principles of the well-established DnCNN NN model, which is widely recognized in the domain of image denoising [31]. This model features a scalable single-pass architecture and comprises four layers categorized into input, intermediate, and output layers. This structural configuration distinguishes it from other NN models utilized for image denoising tasks as it does not incorporate pooling layer operations. Consequently, the receptive field of the PACE-Net model, with a depth of *d*, can be expanded to $\left(\begin{array}{c} 2d+1 \end{array}\right)\times \left(\begin{array}{c} 2d+1 \end{array}\right)$. This enlarged receptive field enables the network to fully leverage contextual information from a broader two-dimensional matrix region. During the training phase, the NN model processes noisy–clean data pairs as inputs. The noisy data serve as the data to be optimized by the NN, while the clean data function as the labels and optimization targets. Specifically, the model takes the rough channel estimation matrix produced by the LS channel estimation algorithm as input and outputs a denoised high-precision channel matrix. The initial layer of the network employs 256 convolutional kernels, each measuring 3×3, to generate 256 feature maps.

**Figure 4:**
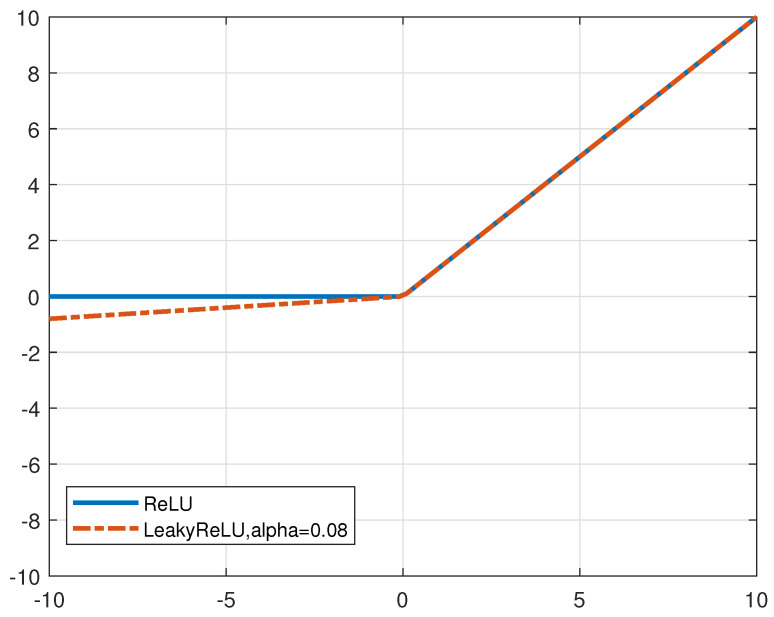
ReLU and LeakyReLU activation function curves.

Figure 4 illustrates the distinctions between the Rectified Linear Unit (ReLU) activation function and the Leaky Rectified Linear Unit (LeakyReLU) activation function, as observed through their respective function curves. Unlike images, where all pixel values are positive, the channel matrix elements can contain a significant number of negative values. The application of the ReLU nonlinear activation function causes these negative values to be set to zero, resulting in the occurrence of phenomena that can adversely affect the convergence of the model. To address this challenge, the PACE-Net NN model extensively employs the LeakyReLU nonlinear activation function as a substitute for the standard ReLU activation function, thereby effectively managing nonlinear features.

A hidden layer consisting of two layers is included in PACE-Net. Features are extracted utilizing 1 × 3 and 3 × 1 convolutional kernels, respectively. Following the feature extraction process, a dropout operation and a LeakyReLU nonlinear activation function are incorporated to facilitate the network’s ability to learn more complex features. Additionally, an attention module is integrated subsequent to the activation function to enhance the network’s feature extraction capabilities further. The final layer of the network is designated as the output layer, which is also a convolutional layer utilizing a 3 × 3 convolutional kernel. This layer is responsible for reconstructing the features extracted by the network into a two-dimensional matrix output.

Residual learning is a methodology designed to address the issue of performance degradation in NN models during the training process [32]. This approach often utilizes a skip connection structure to restore complete data information by thoroughly comparing the information disparity between the residual mapping and the original mapping. By adopting this residual learning strategy, deeper NNs can be trained more effectively, alleviating the phenomena of “gradient disappearance” and “gradient explosion” associated with the deepening of network layers and enhancing overall network performance. The PACE-Net model introduced in this paper presents a novel approach to residual learning. Unlike conventional residual NNs, which typically consist of numerous residual blocks characterized by jump connection structures, PACE-Net conceptualizes the entire model as a single residual block focused on predicting residual data. Specifically, the network is engineered to extract features related to noise information rather than the exact channel matrix information features after noise removal. By employing this approach, it is possible to effectively reduce the number of model layers, decrease the number of parameters, and achieve model lightweighting. The input rough channel matrix image, obtained through LS estimation, is subsequently employed to subtract the noise information features extracted by the network. This process facilitates an accurate estimation of the channel matrix by mitigating the effects of errors and noise.

To enhance the convergence speed of the NN model, improve its convergence efficacy, and prevent overfitting, contemporary research frequently integrates batch normalization operations within the nonlinear activation functions of each layer of the NN [33]. This technique mitigates the effects of internal covariate shift by normalizing input data and stabilizing feature distribution. However, this study observes that the phenomenon of overfitting is more pronounced due to the relatively limited information and richness of channel data compared to traditional image data, in conjunction with the simplicity of feature extraction. As a result, the somewhat indirect nature of batch normalization appears to be less effective in alleviating overfitting and enhancing model convergence. In contrast, the dropout operation addresses the dependencies among neurons by randomly omitting them, thereby promoting the ability of the network to develop more independent and discriminative feature representations. This methodology can stabilize the training process in a direct and robust manner, enhance the generalization capabilities of the model, and decrease the likelihood of overfitting. Consequently, this paper integrates the dropout operation subsequent to the nonlinear activation function of the model to expedite the process of network training, augment the expressive capacity of the network, improve convergence, and decrease sensitivity to network initialization.

To further enhance the feature extraction capabilities of PACE-Net, the polarized self-attention (PSA) mechanism is introduced [34]. The PSA mechanism, originally proposed for computer vision applications, is designed for fine-grained pixel-level tasks. It is characterized by the orthogonal distribution of channel and spatial attention mechanisms, thus facilitating high channel resolutions and high spatial resolutions. Simultaneously, it compresses the input tensor along the corresponding dimensions to reduce the number of model parameters. This enables the NN model to effectively capture high-resolution input/output features and long-range dependency relationships with minimal computational overhead. Thereby, it enables the estimation of highly nonlinear pixel semantics. The structure of the PSA mechanism comprises two branches: one for self-attention in the channel dimension and another for self-attention in the spatial dimension. These two branches can be organized in either a serial or parallel configuration.

**Figure 5:**
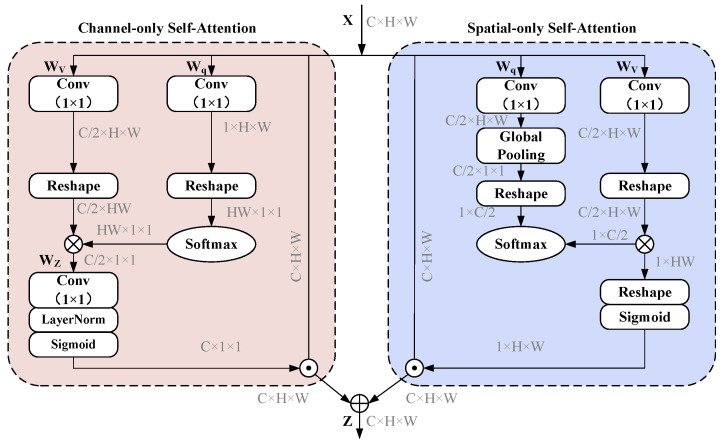
The polarized self-attention (PSA) block under the parallel layout.

**Figure 6:**
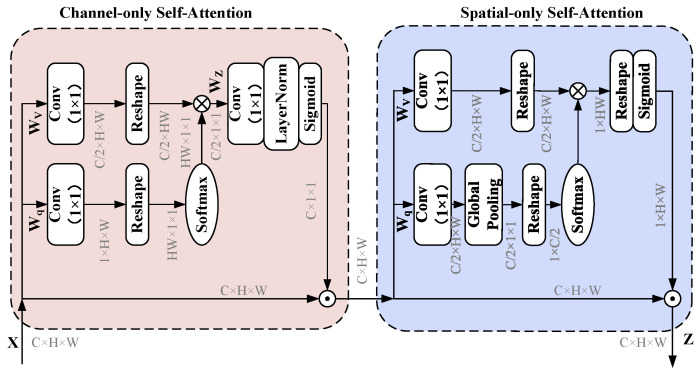
The polarized self-attention (PSA) block under the sequential layout.

The schematic representation of the PSA mechanism is illustrated in Figure 5 and Figure 6. It draws inspiration from the filtering properties of optical lenses to develop polarization filters that selectively target features along a specific dimension (e.g., the channel dimension, which denotes the length of the feature vector at each spatial location), resulting in their complete collapse while preserving the dimensions orthogonal to that direction (e.g., the spatial dimension, which refers to the height and width of the feature map, specifically the spatial distribution within the feature map) at a high resolution. This filtering operation represents a lower degree of information compression, which retains a greater amount of critical information. Consequently, this approach aims to optimize model performance while minimizing the number of computational parameters. To address the potential loss of feature information due to compression in the polarization filtering operation and effectively accommodate fine-grained regression outcomes, the proposed nonlinear enhancement technique incorporates the PSA mechanism. By integrating concepts from High Dynamic Range (HDR) technology in optical imaging, a nonlinear enhancement function that combines Softmax and Sigmoid is employed in both the channel self-attention and spatial self-attention branches. Initially, the Softmax function is applied to the smallest tensor within the attention mechanism module to enhance the dynamic range of attention. Subsequently, the Sigmoid function is utilized for dynamic mapping.

### 4.3. Dataset

The research findings in the field of computer vision suggest that deep artificial NNs offer significant advantages and exhibit outstanding performance in the processing of two-dimensional data. Consequently, this paper aims to transform channel information into a two-dimensional matrix format to generate a dataset. This dataset will serve as input for training and evaluating the performance of PACE-Net.

It is important to note that the channel matrix is a complex matrix in which each element is a complex number, containing both real and imaginary parts. However, NNs struggle to handle complex data directly for several reasons, including the following:

(1) The current research lacks nonlinear activation functions specifically designed for complex data. Directly extending real activation functions to the complex domain may result in gradient vanishing, gradient explosion, or an unstable training process.

(2) The training of NNs relies on the backpropagation algorithm, which requires the calculation of gradients and the updating of parameters. In the complex domain, gradient computation becomes more intricate because the derivatives of complex functions must satisfy the Cauchy–Riemann equations, thereby increasing the complexity of gradient calculations.

(3) Despite various research efforts to develop complex NNs, current deep learning frameworks are primarily designed for real data and offer limited support for complex operations.

Therefore, the channel matrix is decomposed based on its real components, transforming the matrix H into the form $\left\{\begin{array}{cc} Re(H) & -Imag(H)\\ Imag(H) & Re(H) \end{array}\right\}$ through a symmetric mapping technique. This transformation converts the complex matrix of size N×N into a real-valued matrix of size 2N×2N, where *N* denotes the number of antennas at both the receiving and transmitting ends. The current literature predominantly employs the method of converting the channel matrix into a two-dimensional grayscale image. However, this approach presents a significant drawback: since the pixel values in a grayscale image are inherently non-negative, the conversion process necessitates taking the absolute values of any negative elements within the channel matrix, resulting in the loss of critical channel information. In contrast, this paper proposes the direct utilization of the two-dimensional channel matrix as input for PACE-Net. Following the processing of the channel matrix through PACE-Net, the output is subsequently merged using an inverse operation, thereby reconstructing the N×N two-dimensional complex matrix of the same dimensions as the original channel matrix. The training process for PACE-Net involves a data pair comprising the ideal clean channel matrix and the noisy channel matrix derived from LS estimation. The ideal clean channel matrix serves as the label and optimization target, while the noisy channel matrix obtained through LS estimation comprises the data subject to optimization and correction by PACE-Net. During the testing phase, only the noisy channel matrix derived from LS estimation is utilized as input. The training dataset consists of 10,000 data pairs generated using the system model established in Section 1 of this paper, with the validation set containing 3000 data pairs and the test set comprising 1000 noisy channel matrices obtained from LS estimation.

## 5. Computational Complexity Analysis

This section presents a comparative analysis of the computational complexity associated with various channel estimation algorithms. The initial focus is on traditional channel estimation methods, specifically the classical LS channel estimation algorithm and MMSE channel estimation algorithm. For the classical LS channel estimation algorithm, the primary operation involves multiplying a matrix of dimensions $N_{t}N_{r}\times N_{t}N_{r}$ with a vector of dimensions $N_{t}N_{r}$. Consequently, the computational complexity of this algorithm is $O(N_{t}^{2}N_{r}^{2})$. In contrast, the MMSE channel estimation algorithm encompasses several steps, including the estimation of the channel covariance matrix and the computation of the MMSE estimate. The computational complexity of the MMSE algorithm is $O\left(N_{t}^{3}N_{r}^{3}\right)$.

The DL-assisted channel estimation algorithm utilized in this paper initially undergoes an LS channel estimation step. When integrated with the LS channel estimation algorithm, the computational complexity of the LS channel estimation is characterized by $O(N_{t}^{2}N_{r}^{2})$. Subsequently, this estimation is processed through a deep NN, which demonstrates a computational complexity of $O(\sum_{l=1}^{D}C_{in}C_{out}K_{l}^{2}N_{t}N_{r})$. In this context, $C_{in}$ and $C_{out}$ denote the number of input and output channels of the NN, respectively. In this study, both the input and output data are represented as two-dimensional matrices, resulting in both the number of input and output channels being equal to one. Here, *l* is a layer of the NN, *D* signifies the depth of the NN, and $K_{l}^{2}$ indicates the area of the convolution kernel. Consequently, the computational complexity of the proposed PACE-Net can be expressed as $O\left(DK_{l}^{2}N_{t}N_{r}\right)$. To facilitate a more intuitive comparison of the computational complexities associated with various channel estimation algorithms, a summary of these complexities is presented in Table 1.

(1) When $DK_{l}^{2}<N_{t}N_{r}$, the computational complexity of the LS channel estimation algorithm exceeds that of the PACE-Net model. In this context, the computational complexity of the LS+PACE-Net channel estimation algorithm is comparable to that of the LS channel estimation algorithm.

(2) When $DK_{l}^{2}<N_{t}N_{r}<N_{t}^{2}N_{r}^{2}$, the computational complexity of PACE-Net is greater than that of the LS channel estimation algorithm while remaining lower than that of the MMSE channel estimation algorithm. In this context, the computational complexity of the LS+PACE-Net channel estimation algorithm is expressed as $O(DK_{l}^{2}N_{t}N_{r})$, which is consistent with the complexity of PACE-Net.

(3) When $DK_{l}^{2}\geq N_{t}^{2}N_{r}^{2}$, the computational complexity of PACE-Net surpasses that of both the LS channel estimation algorithm and the MMSE channel estimation algorithm. Specifically, the computational complexity of the LS+PACE-Net channel estimation algorithm is equivalent to that of PACE-Net, which is expressed as $O(DK_{l}^{2}N_{t}N_{r})$.

The complexity of each algorithm is analyzed in detail below, accompanied by specific examples. In the DNN model proposed in this study, the parameters are defined as follows: D=4, with both the input and output layers having $K_{in}^{2}=K_{out}^{2}=9$, and the intermediate layer characterized by $K_{middle}^{2}=6$. Consequently, the product $DK_{l}^{2}=24$. Assuming that the number of antennas at both the transmitting and receiving ends of the system is $N_{t}=N_{r}=16$, it follows that $N_{t}N_{r}=256$ and $N_{t}^{2}N_{r}^{2}=65,536$. This scenario falls under the condition where $DK_{l}^{2}<N_{t}N_{r}$. In this case, the LS channel estimation algorithm exceeds that of PACE-Net. The computational complexity of the LS+PACE-Net channel estimation algorithm is equivalent to that of the LS algorithm. Furthermore, the computational complexity of the LS+PACE-Net channel estimation algorithm is significantly lower than that of the MMSE channel estimation algorithm.

It is essential to highlight that the computational complexity of the LS+PACE-Net channel estimation algorithm is lower than that of the traditional MMSE algorithm due to the relatively limited number of layers in the PACE-Net model. Furthermore, the implementation of an offline training and online testing methodology facilitates the majority of the computational complexity associated with training to be executed offline. This strategy effectively optimizes computational resources and training time during the offline phase, leading to a significant decrease in the computational complexity of the online testing model. As a result, the overall computational complexity of the testing model is markedly reduced.

## 6. Simulation Analysis

This section presents the simulation results of the proposed channel estimation algorithm, which is based on PACE-Net. The simulation assumes that both the transceiver and receiver ends of the system are configured in uniform rectangle arrays, and the conduction matrix utilized is a discrete Fourier matrix. The channel is generated in accordance with the Kronecker channel model, as characterized by Equation (4), while the correlation matrix is derived based on Equation (5), utilizing the specific parameters detailed in Table 2.

NMSE is employed in the simulation to evaluate the performance of the algorithm, and it is defined as follows:

(10) $$\mathrm{NMSE}=E\left[10\log_{10}\left({∥\hat{h}-h∥}_{2}^{2}/{∥h∥}_{2}^{2}\right)\right],$$

where h represents the true channel, while $\hat{h}$ denotes the estimated channel.

The proposed LS+PACE-Net channel estimation algorithm (denoted as “LS+PACE-Net”) is compared with LS-based channel estimation algorithm (denoted as “LS”), MMSE algorithm (denoted as “MMSE”), and the existing DL-assisted algorithm utilizing DnCNN NN model (denoted as “LS+DnCNN”) in the simulation.

The training loss function for the PACE-Net model was selected to be the mean squared error loss, which is defined as follows:

(11) $$l\left(\Theta \right)=E\left[{∥R\left(z_{i};\Theta \right)-\left(z_{i}-x_{i}\right)∥}_{F}^{2}\right]$$

where Θ represents the parameters of the network, while the notation ${\left\{\left(z_{i},x_{i}\right)\right\}}_{i=1}^{N}$ signifies the N pairs of noisy training data, and ${\left\{\left(z_{i},x_{i}\right)\right\}}_{i=1}^{N}$ denotes the input–output function of the NN. The specific configuration of the network training parameters is detailed in Table 3.

**Figure 7:**
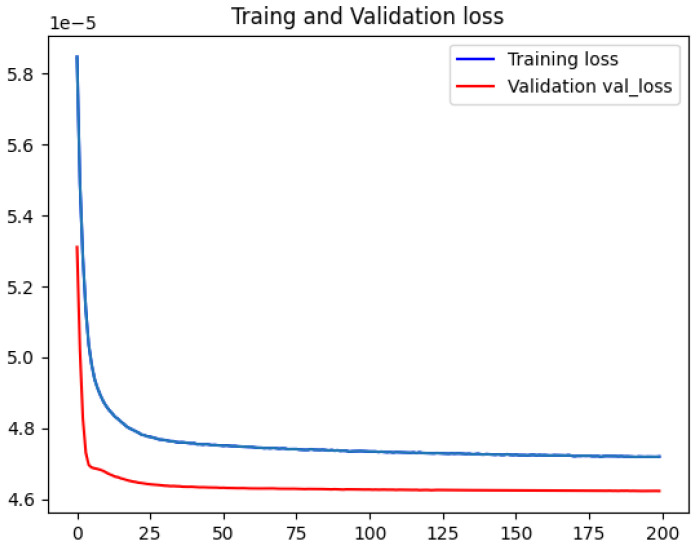
Loss function change curve of training set/validation set during model training process.

Additionally, the convergence of the model throughout the training process is illustrated in Figure 7. It is evident from the figure that the model exhibits satisfactory convergence, and the trajectory of the loss function curve demonstrates a relatively smooth progression.

**Figure 8:**
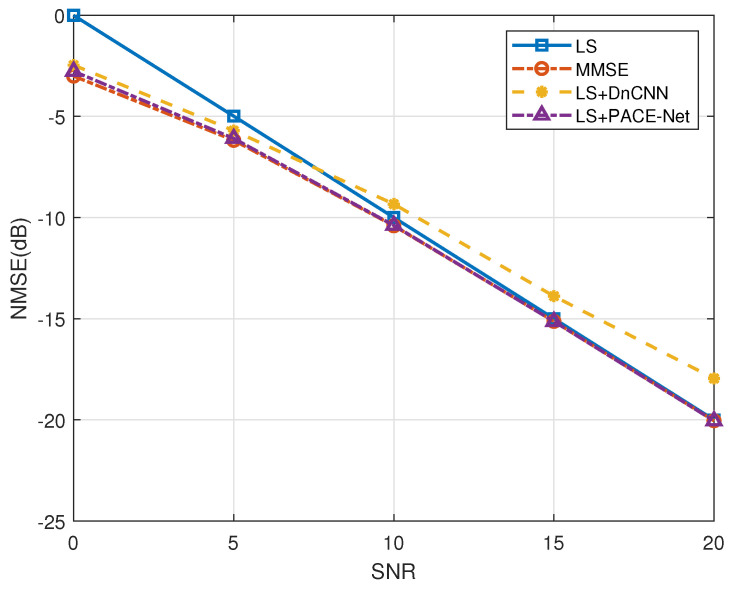
Comparison of NMSE performance of different algorithms under independent channel.

Figure 8 illustrates the NMSE as a function of the SNR for various algorithms under the condition that the antennas operate independently. The data presented in Figure 8 indicate that the LS+PACE-Net algorithm proposed in this study demonstrates a significant performance advantage over the traditional LS algorithm, particularly when the SNR is below 5 dB. Specifically, the NMSE performance of the LS+PACE-Net algorithm is enhanced by approximately 4 dB compared to the LS algorithm, and its NMSE performance approaches that of the conventional MMSE channel estimation algorithm. Furthermore, in comparison to the existing DnCNN NN model, the PACE-Net model introduced in this paper exhibits an NMSE performance improvement of approximately 0.3 dB under low-SNR conditions. As the SNR increases, the performance advantage of the PACE-Net model becomes increasingly pronounced, achieving a maximum NMSE performance enhancement of about 3 dB relative to the DnCNN model when the SNR exceeds 5 dB.

**Figure 9:**
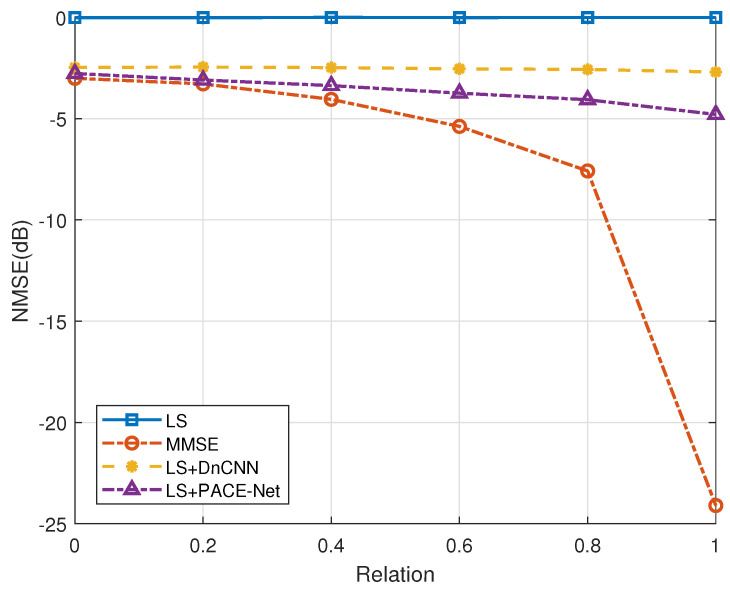
Effect of antenna correlation on the performance of each algorithm, SNR = 0 dB.

**Figure 10:**
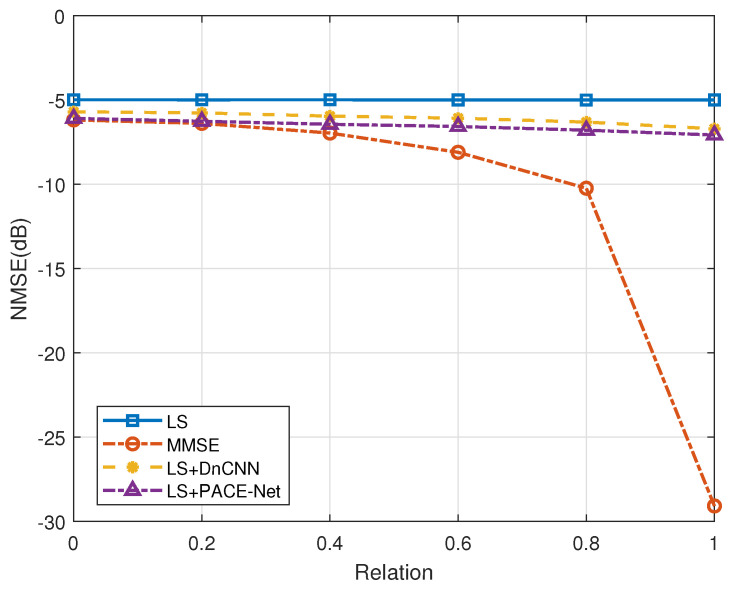
Effect of antenna correlation on the performance of each algorithm, SNR = 5 dB.

Secondly, the performance of various channel estimation algorithms is evaluated in the context of antenna correlation. Figure 9 and Figure 10 illustrate the performance curves of the different algorithms in relation to antenna correlation at varying SNR ratios. The degree of antenna correlation is manipulated by adjusting the parameter *a*, which ranges from 0 to 1. As depicted in these figures, the LS channel estimation algorithm demonstrates minimal sensitivity to antenna correlation. In contrast, the NMSE performance of the LS+DnCNN and LS+PACE-Net channel estimation algorithms exhibits a slight improvement with increasing antenna correlation. However, this improvement is marginal. Conversely, the NMSE performance of the MMSE channel estimation algorithm is significantly influenced by antenna correlation, with a more pronounced improvement in NMSE performance as antenna correlation increases. Notably, when the antenna correlation is below 0.2, the NMSE performance of the LS+PACE-Net channel estimation algorithm proposed in this study is nearly equivalent to that of the MMSE channel estimation algorithm. However, as antenna correlation rises, the NMSE performance of the MMSE algorithm improves at a faster rate, thereby amplifying its comparative advantage.

The performance of the MMSE channel estimation algorithm exhibits a significant enhancement when the antenna correlation coefficient is denoted as a=1. This improvement can be attributed to the low-rank characteristic of the channel covariance matrix under the condition a=1, where the rank is considerably lower than the dimensions of the channel covariance matrix. The existing literature [35,36] indicates that the theoretical estimation performance of the MMSE estimator can achieve substantial gains when the channel covariance matrix possesses a low-rank structure. In contrast, other algorithms fail to attain this performance upper bound due to the challenges associated with accurately reconstructing the low-rank property. It is essential to note that the scenario where a=1, indicating perfect correlation among all antennas, is primarily a theoretical construct and holds limited practical applicability.

**Figure 11:**
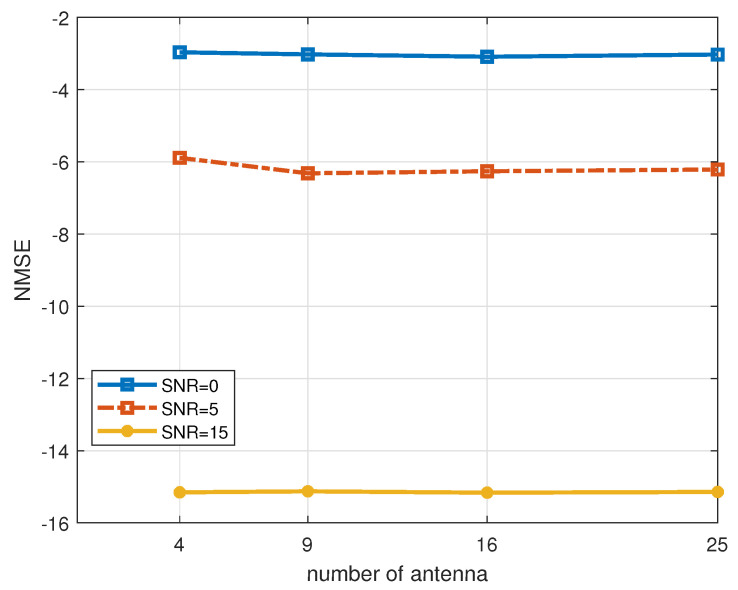
Effect of the number of antennas on the performance of the algorithm, a=0.2.

Figure 11 provides an analytical assessment of the influence of varying antenna counts on the channel estimation algorithm proposed in this study. The simulation maintains a constant number of antennas on both the receiver and transmitter sides, with SNRs fixed at 0 dB, 5 dB, and 15 dB, and an antenna correlation coefficient of a=0.2. As illustrated in Figure 11, the number of antennas appears to exert a negligible impact on the NMSE performance of the proposed algorithm.

## 7. Ablation Experiment

**Figure 12:**
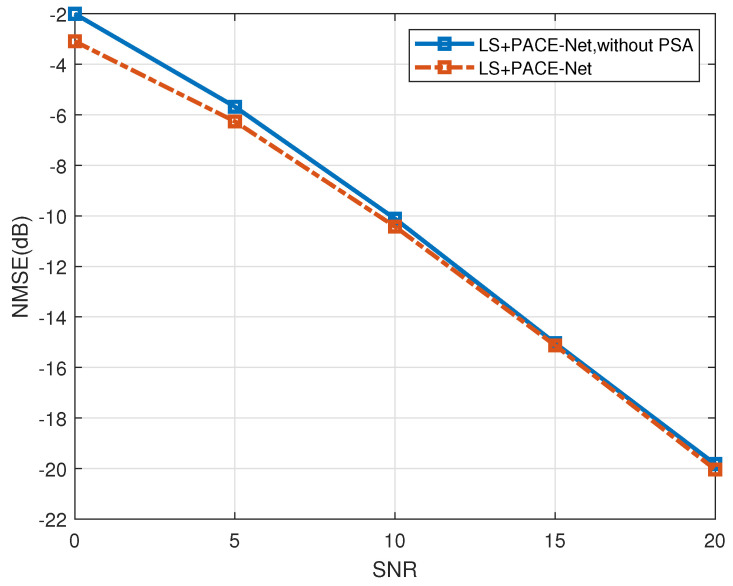
PSA module performance validation, a=0.2.

This section evaluates the efficacy of each innovative component of the PACE-Net model proposed in this paper through the implementation of ablation experiments. Figure 12 shows the evaluation of the effectiveness of the PSA mechanism. The LS+PACE-Net channel estimation algorithm, which excludes the PSA mechanism, is selected as the comparative algorithm (denoted as “LS+PACE-Net, WITHOUT PSA”). The antenna correlation is set to a=0.2 during the simulation.

As illustrated in Figure 12, the incorporation of the PSA mechanism into the channel estimation algorithm enhances its performance compared to the algorithm that does not utilize the PSA mechanism. Specifically, under low-SNR conditions, the performance improvement reaches a maximum of approximately 2 dB, while, under high-SNR conditions, the enhancement is about 0.5 dB. These results suggest that the PSA mechanism significantly enhances the capability to extract and mitigate noise bias of the NN model, thereby improving the overall performance of the algorithm. The simulation results demonstrate the effectiveness of the PSA mechanism.

**Figure 13:**
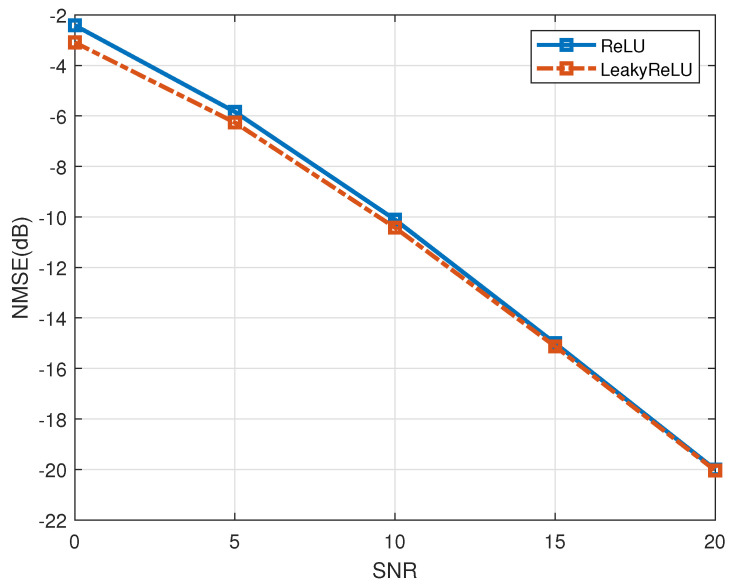
Performance validation of LeakyReLU activation function, a=0.2.

In the following analysis, Figure 13 shows the evaluation results of the LeakyReLU activation function. As previously noted, channel matrix data, in contrast to image data, include negative elements. The implementation of the LeakyReLU activation function effectively mitigates the occurrence of “dead neurons” and ensures that the model converges efficiently compared to the traditionally utilized ReLU activation function. The proposed algorithm is the LS+PACE-Net channel estimation algorithm utilizing the LeakyReLU activation function (denoted as “LeakyReLU”), and the comparative algorithm is the LS+PACE-Net channel estimation algorithm utilizing the ReLU activation function (denoted as “ReLU”) with the antenna correlation set at 0.2. As depicted in Figure 13, the NMSE performance of the LS+PACE-Net algorithm utilizing the LeakyReLU activation function demonstrates an improvement of approximately 0.6 dB when compared to the LS+PACE-Net algorithm employing the ReLU activation function. This finding substantiates the effectiveness of the LeakyReLU activation function in enhancing the performance of the LS+PACE-Net channel estimation algorithm.

**Figure 14:**
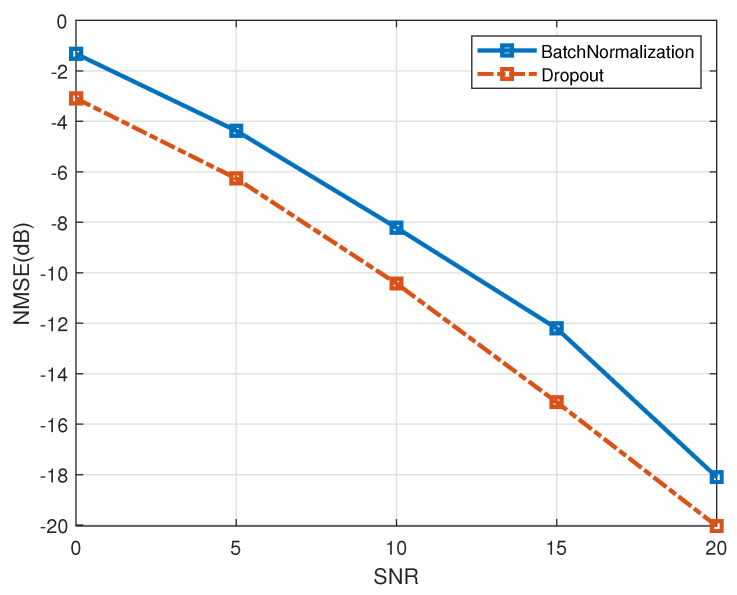
Verification of dropout performance, a=0.2.

Finally, the effectiveness of the dropout operation has been evaluated. The simulation results are presented in Figure 14. The comparative algorithm utilized is the LS+PACE-Net channel estimation algorithm, which substitutes the dropout operation (denoted as “dropout”) with the BatchNormalization operation (denoted as “BatchNormalization”). The antenna correlation is set to 0.2. The data illustrated in the figure indicate that the NMSE performance of the LS+PACE-Net algorithm employing the dropout operation exhibits an improvement of approximately 3 dB when contrasted with the LS+PACE-Net algorithm utilizing the BatchNormalization operation. This finding suggests that the dropout operation is more advantageous for the training of the NN model proposed in this study, leading to enhanced model performance. Consequently, this validates the effectiveness of incorporating the dropout operation.

## 8. Conclusions

In this paper, we investigate a DL-assisted intelligent channel estimation algorithm tailored for massive MIMO systems. We reformulate the conventional channel estimation approach as an image denoising problem and propose the PACE-Net NN model to achieve this objective. The PACE-Net model utilizes a data-driven approach, incorporating online training and offline testing methodologies. Additionally, PACE-Net enhances the feature extraction capability of the model through joint residual learning, a polarized self-attention mechanism, the LeakyReLU nonlinear activation function, and dropout operations. These enhancements contribute to improved image denoising performance and channel estimation accuracy while simultaneously reducing computational complexity and reliance on channel sparsity. Ultimately, the simulation results demonstrate that the estimation accuracy of the PACE-Net-assisted intelligent channel estimation algorithm proposed in this study significantly surpasses that of the traditional LS algorithm and the DnCNN-assisted intelligent channel estimation algorithm. Furthermore, under certain conditions, its performance is comparable to that of the traditional MMSE algorithm while exhibiting substantially lower computational complexity than the traditional MMSE channel estimation algorithm.

## Figures and Tables

**Table 1:** Computational complexity of different channel estimation algorithms.

Channel Estimation Methods	Computational Complexity
LS Channel Estimation Algorithm	$O\left(N_{t}^{2}N_{r}^{2}\right)$
MMSE Channel Estimation Algorithm	$O\left(N_{t}^{3}N_{r}^{3}\right)$
PACE-Net model	$O\left(DK_{l}^{2}N_{t}N_{r}\right)$

**Table 2:** Configuration of channel model parameters for massive MIMO communication system.

Parameter	Value
Type of channel fading	Rayleigh
Number of transmit antennas	16
Number of receive antennas	16
Frequency of carrier wave/GHz	15
The speed of light/(m/s)	$3\times 10^{8}$
Communication Distance/m	1000
Reference Distance/m	100
Path Decay Factor	3
Number of pilots	16

**Table 3:** Configuration of PACE-Net training parameters.

Parameter	Value
SNR/dB	0, 5, 10, 15, 20
Learning Rate	$10^{-5}$
BatchSize	256
Epoch	200
Optimizer	Adam
Programming Framework	Keras
GPU	Nvidia Titan 3080

## Data Availability

All data generated or analyzed during this study are included in this published article.
